# Spatiotemporal dynamics of high-wavenumber turbulence in a basic laboratory plasma

**DOI:** 10.1038/s41598-022-23559-1

**Published:** 2022-12-12

**Authors:** Yuichi Kawachi, Makoto Sasaki, Yusuke Kosuga, Kenichiro Terasaka, Takashi Nishizawa, Takuma Yamada, Naohiro Kasuya, Chanho Moon, Shigeru Inagaki

**Affiliations:** 1 National Institute for Fusion Science, National Institutes of Natural Sciences, Toki, Gihu 509-5292 Japan; 2 College of Industrial Technology, Nihon University, Narashino, Chiba 275-8575 Japan; 3 Research Institute for Applied Mechanics, Kyushu University, Kasuga, Fukuoka 816-8580 Japan; 4 Research Center for Plasma Turbulence, Kyushu University, Kasuga, Fukuoka 816-8580 Japan; 5 Interdisciplinary Graduate School of Engineering Sciences, Kyushu University, Kasuga, Fukuoka 816-8580 Japan; 6 Faculty of Arts and Science, Kyushu University, Motooka, Fukuoka 819-0395 Japan; 7 Institute of Advanced Energy, Kyoto University, Uji, Kuoto 611-0011 Japan

**Keywords:** Plasma physics, Magnetically confined plasmas

## Abstract

High-spatial resolution observation of high-wavenumber broadband turbulence is achieved by controlling the magnetic field to be relatively low and measuring with a azimuthally arranged multi-channel Langmuir array in a basic laboratory plasma. The observed turbulence consists of narrowband low-frequency fluctuations and broadband high-frequency turbulent fluctuations. The low-frequency fluctuations have a frequency of about 0.7 times the ion cyclotron frequency and a spatial scale of 1/10 of the ion inertial scale. In comparison, high-frequency fluctuations have a higher frequency than the ion cyclotron frequency and spatial scales of 1/10–1/40 of the ion inertial scale. Two-dimensional correlation analysis evaluates the spatial and temporal correlation lengths and reveals that the high-wavenumber broadband fluctuations have turbulent characteristics. The measurements give us further understanding of small scale turbulence in space and fusion plasmas.

## Introduction

High-wavenumber or small-scale turbulence is believed to play an essential role in anomalous electron energy transport, anomalous heating, and particle energization in space and fusion plasmas^1–7^. Here the “small”-scale turbulence means turbulence with a smaller scale than the ion inertial scale length $\rho _\mathrm {s}$, i.e. characterized as $k_\perp \rho _\mathrm{s} \ge 1$ including sub ion scale $k_\perp \rho _\mathrm{s} \ge 1$ and electron scale $k_\perp \rho _\mathrm {e} \gg k_\perp \rho _\mathrm{s} \gg 1$. Here, $\rho _\mathrm {s}=C_\mathrm {s}/\Omega _\mathrm {ci}$, $C_\mathrm {s}$ is the ion sound velocity, $\Omega _\mathrm {ci}$ is the ion cyclotron angular frequency, $k_\perp$ is a typical wavenumber perpendicular to the magnetic field. $\rho _\mathrm {e}=V_\mathrm {th,e}/\Omega _\mathrm {ce}$ is electron Lamor radius, where $V_\mathrm {th,e}$ is electron thermal velocity and $\Omega _\mathrm {ce}$ is electron cyclotron angular frequency. Instances of high-wavenumber turbulence are kinetic Alfvén waves and ion cyclotron waves related to anomalous heating and particle energization in space plasmas^3,4^, and electron temperature gradient modes related to anomalous electron energy transport in fusion plasmas^5,6^.

Observations of the spatiotemporal dynamics of turbulence in basic laboratory plasmas are essential for validation of fundamental theoretical and simulation studies and understanding more complex processes such as wave-trapping, shear-flow decorrelation, and meso-scale flow generation^3–8^. Previously, many studies of ion scale turbulence ($k_\perp \rho _\mathrm{s} \ll 1$) have been elucidated in basic plasma devices, including turbulent transport and structure formation^9–12^. The turbulence characteristics observed in linear plasmas are often common to those of torus plasmas^13,14^, even though some parameters are far apart. However, there have been few experimental studies of high-wavenumber turbulence. Furthermore, there are no examples of detailed observations of their spatiotemporal dynamics. As well as the studies of ion scale turbulence, the detailed observation of the spatiotemporal structure of high-wavenumber turbulence should provide fruitful insights into understanding the fundamental physical processes of high-wavenumber turbulence.

In general, the measurement of high-wavenumber turbulence is challenging because it requires high-spatial resolution. Scattering diagnostics and phase-contrast imaging have been proposed for high-wavenumber fluctuation measurement in torus plasmas^15–17^ and in some linear plasmas^18^. Scattering diagnostics allow us local measurements with high-spatial resolution, but they are difficult for simultaneous multi-point and multi-wavenumber measurement. Although the phase-contrast imaging can evaluate a two-dimensional wavenumber spectrum, reconstruction of local fluctuations requires additional analyses because it is line integrated measurement. Therefore, it is not sufficient for measuring spatiotemporal dynamics of high-wavenumber turbulence in detail. Essentially, basic laboratory plasmas are more suitable for studying spatiotemporal dynamics of high-wavenumber turbulence, because Langmuir probes with a high signal-to-noise ratio, high-spatial resolution, and scalability for multipoint measurement, can be utilized at low temperature. However, there are few examples of observation of high-wavenumber turbulence by Langmuir probes^19,20^. Moreover, the spatial structure of high-wavenumber fluctuations is studied only by two-point correlation analyses. Azimuthally aligned probe arrays have been used to study ion-scale turbulence with fundamental modes up to m=5^21–23^. Although it potentially has high enough spatial resolution to measure sub-ion and electron scale turbulence, have not been utilized to observe these scale turbulence.

In this article, we present the first experimental observation of spatiotemporal dynamics of high-wavenumber turbulence, which is achieved by controlling ion inertial scale and high-spatial resolution measurement by an azimuthally equal-aligned multi-channel Langmuir probe array. Two-dimensional spectral analysis shows the coexistence of narrowband and broadband turbulent components of high-wavenumber turbulence. Moreover, the space-time two-dimensional autocorrelation analysis reveals the coherent nature of narroband fluctuations and the turbulent natuer of the broadband flucution. Both fluctuations rotate in an electron diamagnetic direction with a different dispersion relation and correlation length. These detailed spatiotemporal dynamics measurements are pioneering work for studying the fundamental process of high-wavenumber turbulence in a basic laboratory plasma.

## Results

### Experimental condition

We investigate the spatiotemporal dynamics of high-wavenumber turbulence with $k_\theta \rho _\mathrm {s}\ge 1$ on the PANTA(Methods), by controlling the magnetic field to a relatively low $B_\mathrm {ax}=$ 22.5 mT. In the lower magnetic field, the ion inertial scale length $\rho _\mathrm {s}$ is about 40 mm. We note that the ion inertial scale is small enough to be less than the distance between the vacuum wall and plasma that there is no lost ion to the wall. The larger $\rho _\mathrm {s}$ has the following two advantages in the measurement of high-wavenumber turbulence. The first is to extend the range of a normalized wavenumber which can be measured with the 64ch probe array (Methods). In other words, the measurable range of normalized wavenumbers is $k_\theta \rho _\mathrm {s} \approx$ 1–32 at 22.5 mT, whereas it is $k_\theta \rho _\mathrm {s} \approx$ 0.25–8 at $B_\mathrm {ax}=$ 90 mT. The other is that the fluctuation scale to be excited can be controlled, because the excited fluctuations should satisfy periodic boundary conditions in the azimuthal direction. Specifically, only a fluctuation of $k_\theta \rho _\mathrm {s} \ge 1$ can be excited at the lower magnetic field. Thus, the objective of the experimental conditions aims to observe the spatiotemporal dynamics from sub ion scale to electron scale ($k_\theta \rho _\mathrm {e} \gg k_\theta \rho _\mathrm{s} \ge 1$) by the 64ch probe array.

**Figure 1:**
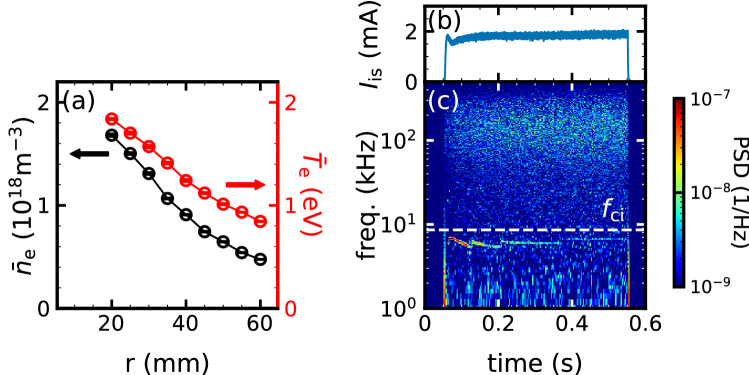
(**a**) Radial profiles of electron density (black) and electron temperature (red). Time evolution of (**b**) ion saturation current and (**c**) frequency spectrum. White dashed line in (**c**) indicates ion cyclotron frequency.

Typical plasma profiles in the experiments are shown in Fig. 1a. The peaked profiles of high electron density and low electron temperature are formed with central electral density of $n_\mathrm {e} \approx 2\times 10^{18}/\mathrm {m^{3}}$ and electron temprature of $T_\mathrm {e} \approx 2\,\mathrm {eV}$, respectively. The density gradient scale length is about $L_n \sim 50$ mm which is the same order of $\rho _\mathrm {s}$. Since $\rho _\mathrm {s}/L_n \sim 1$, the diamagnetic drift velocity $V_*$ is almost the same as the ion sound speed, i.e. $V_*=C_\mathrm {s}\rho _\mathrm {s}/L_n\approx C_\mathrm {s}=\sqrt{eT_\mathrm {e}/m_\mathrm {i}} \sim 2000\,\mathrm {m/s}$. The electron temperature scale length is about $L_n \sim 65$ mm. The ratio of the gradient scale length of density to that of temperature is $\eta _\mathrm {e} \sim 0.77$ The electron density fluctuations are measured from the ion saturation current $I_\mathrm {is}$ as $\tilde{n}_\mathrm {e} \propto \tilde{I}_\mathrm {is}$, assuming the electron temperature fluctuation is negligible^24^. Figure 1b and c show the time evolution of $I_\mathrm {is}$ and the corresponding spectrogram. The total amplitude of the fluctuation normalized to the equilibrium is a few percent, which is small compared to the tens of percent amplitude of the drift waves at the higher magnetic field condition^11,25^. There are two fluctuation components, consisting of lower and higher frequency ones than the ion cyclotron frequency $f_\mathrm {ci} = 8.5\,\mathrm {kHz}$. They are quasi-stationary at 0.24–0.54 s, so subsequent spectral analyses use these periods to evaluate the stationary spectrum.

### Observation of spatiotemporal dynamics of high wavenumber turbulence.

**Figure 2:**
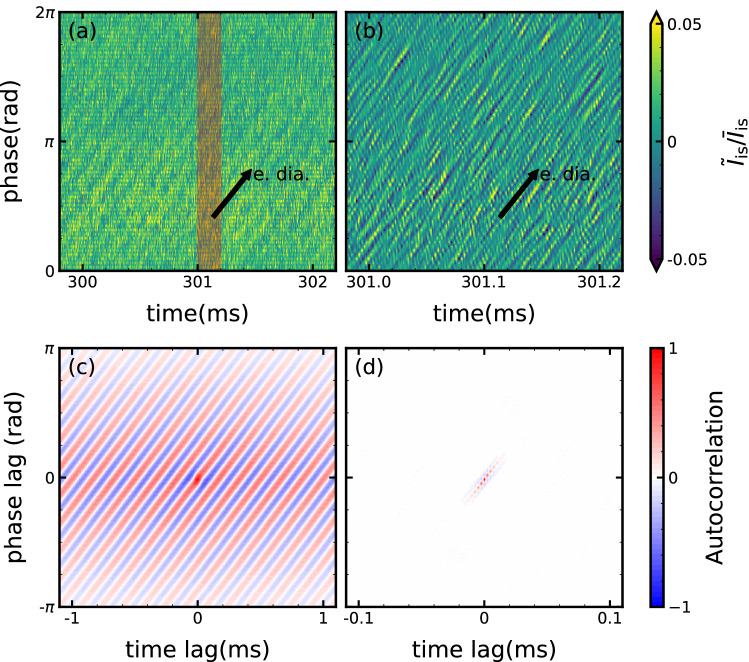
(**a**) Spatiotemporal evolution of normalized ion saturation current. (**b**) An enlarged view of the red shaded area in (**a**). Black arrows in (**a**) and (**b**) indicate propagation direction. Two-dimensional autocorrelation for (**c**) low-frequency component and (**d**) high-frequency component function evaluated from (**a**) and (**b**).

All of the probe tips of the azimuthal 64ch probe array measure the spatiotemporal evolution of normalized density fluctuations, as shown in Fig. 2a. The direction of the increasing azimuthal phase corresponds to the electron diamagnetic direction. It can be seen that the fluctuation corresponding to the lower frequency component propagates in the electron diamagnetic direction with a small spatial structure. Figure 2b shows an enlarged view of Fig. 2a with a time width of 0.25 ms. The many smaller structures corresponding to higher frequency components are identified. They also propagate in the electron diamagnetic direction, but appear to decay as they propagate, which is also confirmed in the following analysis. Thus, as the aim of the experiment, finer scale turbulence was successfully observed by using the full capability of the 64ch probe array.

Figure 2c shows a two-dimensional space-time autocorrelation function (Methods) calculated from the spatiotemporal evolution observed in Fig. 2a. Here a band-pass filter (1–20 kHz) is applied to each time series signal before calculating the two-dimensional autocorrelation function. The correlation structure has a frequency of 6.5 kHz. The azimuthal mode number is $m \approx 10$ which corresponds to $k_\theta \sim 2.5/\mathrm {cm}$. The temporal and spatial correlations are much longer than the fluctuation’s own scale. The spatiotemporal evolution of the peak of the correlation indicates the coherent propagation of the low-frequency fluctuation in the electron diamagnetic direction. The two-dimensional autocorrelation is also applied to the high-frequency fluctuation (20–500 kHz) observed in Fig. 2b, as shown in Figure 2d. The extracted structure has a frequency of about 110 kHz and a wavenumber of $k_\theta \approx 3.7/\mathrm {cm}$. The autocorrelation decays rapidly to 1/*e* within a few mm and a few $\mathrm {\mu s}$, which is a spatially and temporally shorter interval than the fluctuation scales. The peak of autocorrelation propagates in the electron diamagnetic direction, while its value decays to 1/*e* by order of the spatiotemporal scale of the fluctuations.

### Spectral characteristics of high-wavenumber turbulence.

**Figure 3:**
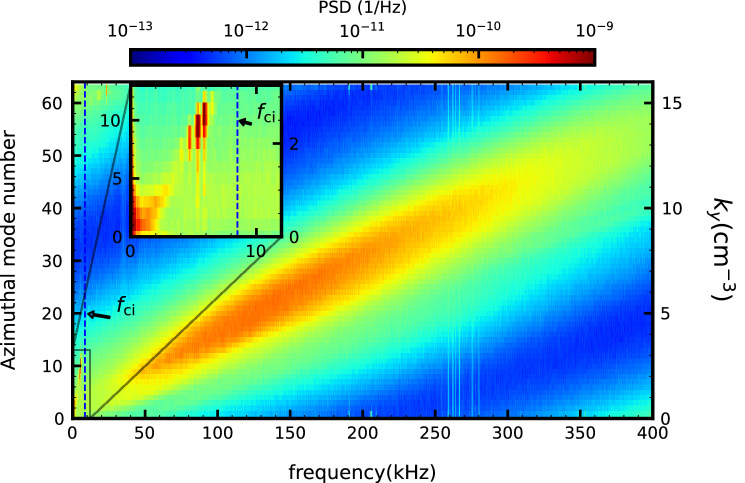
A contour plot of two-dimensional frequency and wavenumber power spectrum density of the normalized ion saturation current observed in Fig. 2. A subfigure in this graphshows an enlarged view of the low-frequency range. Dashed lines indicate the ion cyclotron frequency $f_\mathrm {ci}$. The left and right vertical axes denote the azimuthal mode number *m* and the corresponding wavenumber $k_\theta$, respectively.

The observed spatiotemporal evolution can be used to evaluate the power spectrum density in frequency and wavenumber space by directly calculating the two-dimensional Fourier transform. Figure 3 shows the two-dimensional power spectrum density of normalized density fluctuation in frequency and wavenumber space. Here we assume the fluctuations only propagate in the electron diamagnetic direction, based on the results shown in Fig. 2a–d, that is, the evaluated wavenumber spectrum only allow positive values corresponding to wavenumbers in the electron diamagnetic direction. Narrow peaks are observed around $m=$ 9–11 and $f=$ 5–7 kHz. It is considered that the multiple peaks are caused by nonlinear interaction between the fundamental $m \approx 10$ mode and the lower frequency $m=1$ mode. The phase velocity $v_\mathrm {p}$ is about $v_\mathrm {p}\approx$150 m/s, which is much smaller than the electron diamagnetic drift velocity. Broadband peaks are evident over wide ranges of frequency above $f_\mathrm {ci}$ and the wavenumber. The ranges of frequency and wavenumber are $f=$30–300 kHz and $m=$ 8–40 (corresponding to $k_\theta =$ 2–10/cm), respectively. The broadband peaks are on a linear function in frequency and wavenumber space, and the phase velocity is $v_\mathrm {p} \approx 2000\mathrm {m/s}$, which almost coincides with the electron diamagnetic drift velocity and the ion sound velocity, i.e. $v_\mathrm {p}\approx V_* \approx C_\mathrm {s}$.

**Figure 4:**
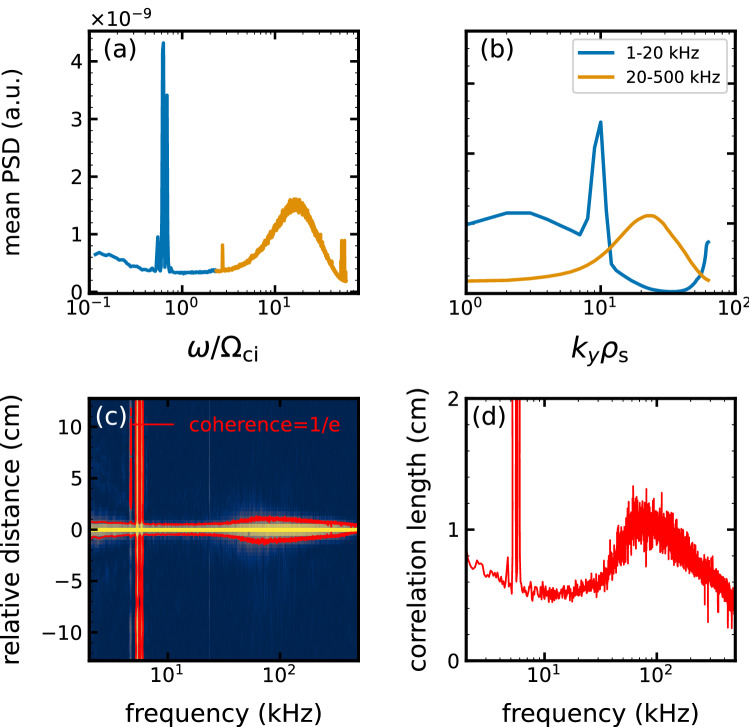
Semi-logarithmic plots of (**a**) normalized frequency $\omega /\Omega _\mathrm {ci}$ spectra and (**b**) normalized wavenumber $k_\theta \rho _\mathrm {s}$ spectra of the normalized ion saturation current. Blue and orange lines indicate the low-frequency (1–20 kHz) and high-frequency (20–500 kHz) components, respectively. (**c**) An azimuthal distribution of squared coherence between one of the probes at $\theta =0$ and the others of 64ch probe array. Red lines in (**c**) and (**d**) shows correlation length spectrum.

The one-dimensional frequency/wavenumber spectrum is evaluated by averaging the low and high-frequency components, as shown in Fig. 4a and b. The frequency is normalized by $f_\mathrm {ci}$ as $f/f_\mathrm {ci}=\omega / \Omega _\mathrm {ci}$, where $\omega$ is the angular frequency, while the wavenumber is normalized by $\rho _\mathrm {s}$ as $k_\theta \rho _\mathrm {s}$. The normalized frequency of the low-frequency fluctuations is about $\omega /\Omega _\mathrm {ci}=$ 0.7 and the normalized wavenumber is $k_\theta \rho _\mathrm {s}\approx$ 10. The broadband high-frequency fluctuations have the normalized frequency of a range of $\omega /\Omega _\mathrm {ci} =$ 10–30 and the normalized wavenumber of a range of $k_\theta \rho _\mathrm {s}=$ 10–40. These results demonstrate successful observations of high-wavenumber turbulence with $k_\theta \rho _\mathrm {s} \gg 1$.

Figure 4c shows an azimuthal distribution of the squared coherence spectrum between the reference signal of the 64ch probe array at $\theta =0$ rad and the other signals as a function of frequency and relative azimuthal distance from the reference probe. From Fig. 4c, the azimuthal correlation length is estimated as the distance from the reference probe at which the correlation decays to 1/*e*, as shown in Fig. 4d. The low-frequency components have a correlation of more than 1/*e* at a distance of 12.5 cm (corresponding to an azimuthal angle $\pi$ rad) in the azimuthal direction. It means that the fluctuation structure propagates coherently, which is consistent with the results of the autocorrelation function analysis in Fig. 2c. The high frequency components have small spatial correlation lengths. The correlation length peaks at f = 60 kHz and its scale is comparable to the wavelength of the broadband fluctuations. Thus, as in the autocorrelation analysis, the spectral analysis indicates the coherent nature of the low-frequency fluctuations and the turbulent characteristics of the high-frequency fluctuations.

## Discussion

Here we summarize and discuss our experimental observations. Firstly, we discuss neutral-plasma collision which is infomative to compare realistic applications, such as edge/SOL turbulence and thruster. Typicaly the linear laboratory plasma could be collisional with strong neutral-electron and neutral-ion interaction. In this experiment, the neutral densityis $\sim \mathrm {10^{20}/m^{3}}$. The collision cross section for neutral and electrons, and for neutral and ions, are $\mathrm {10^{-20}/m^{2}}$ and $\mathrm {10^{-18}/m^{2}}$, respectively. Here we use the electron temperature of 1 eV and ion temperature of 0.1 V. Using the neutral density and the cross section, the neutral-electron collision frequency is evaluated as $\sim$ 400 kHz while the neutral-ion collision freuqency is evaluated as $\sim$ 50  kHz. Namely, both electrons and ions are collisional with neutral particle for fluctuation below 50 kHz. On the other hands, for fluctauations with above 50  kHz, the ions are collisionless and the electrons are collisional in this experiment. Neutral particle effects play an important role in plasma confinement and gas puff fueling in the scrape-off layer(SOL)^26–29^. Our results will contribute to turbulence physics including neutral effetcs in the SOL plasma^30–33^. In addition to this, since the experimental conditions of the present study are in parameters close to those of hall-thrusters^34^, magnetic nozzle thrusters^35^, or ionospheric plasmas^36^, it should impact on these studies.

**Figure 5:**
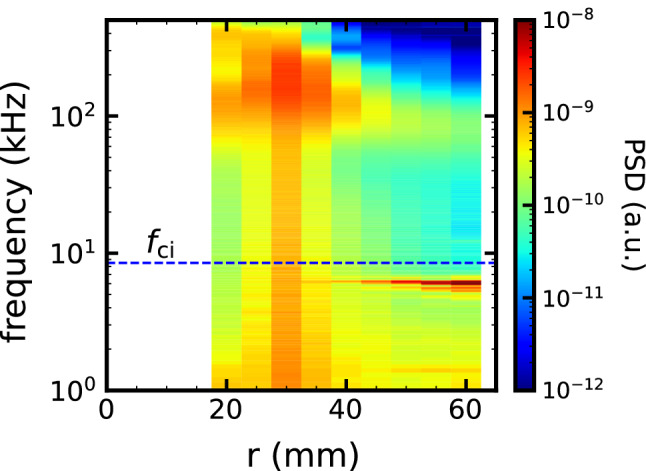
Radial profile of power spectrum density of normalized density fluctuations. Dashed line indicate the ion cyclotron frequency $f_\mathrm {ci}$.

Secondery, we consider to identification of the observed modes. Two types of high-wavenumber fluctuations were observed in this experimental configuration: low and high-frequency fluctuations. The low-frequency one has a time scale of 0.7 times the ion cyclotron frequency and a spatial scale of ten times the ion inertial scale. The ions and electrons moves collisional in this mode. The azimuthal propagation velocity is 150 m/s, which is much slower than the electron diamagnetic drift velocity. Spatial and temporal correlation scales are longer than the fluctuation scale, which indicate that the low-frequency fluctuation propagates coherently in the electron diamagnetic direction. From the point of view of a dispersion relation, kinetic Alfven waves or electromagnetic ion cyclotron mode could be a candidates of the low-frequency modes^37,38^. These modes are important in the ionosphere and magnetosphere, and magnetic fluctuation measurements are necessary to confirm whether these electromagnetic modes destabilizes. Figure 5 shows radial profile of power spectrum density of the density fluctaiotion. Since the low-frequency mode seems to exist at edge region($r=$ 60 mm), the free energy source is not the pressure gradient. At the plasma edge $r=$ 60 mm, neutral particle is richer than core and its effect should be strong. Thus, another candidates of identity of the low freuquency modes could be related neutral pressure gradient from edge to core^30,31,39^.

The high-frequency fluctuations are distributed in wide ranges of frequency and wavenumber. The time and spatial scales are 10–30 times the ion cyclotron frequency and 10–40 times ion inertia length, respectively. The higher frequency compared to the neutral-ion collision frequency indicates that the ions are moving without collision with neutral in the high-frequency fluctuations. The phase velocity is close to the electron diamagnetic velocity and ion sound velocity. The correlation analysis indicates that the high-frequency turbulence has turbulent characteristics.The high-frequency mode is destabilized at $r\le \,$40 mm, as shown Fig. 5. Therefore, the high-frequency mode shold be destabilized by pressure gradient.Candidates for high-wavenumber ($k_\theta \rho _\mathrm {s}\gg 1$) and high-frequency ($f_\mathrm {ci}<f<f_\mathrm {LH}$) electrostatic turbulence driven by pressure gradient include the electron temperature gradient mode (ETG), the electrostatic ion cyclotron wave (EICW) and the ion cyclotron drift wave (ICDW) and so on. Conventional theory suggests that the destabilization of typical ETGs requires a gradient in electron temperature that is stronger than the gradient in density^5^ while whistler ETG mode is destabilized even if the ratio of the scale length is $\eta _\mathrm {e} > 2/3$^40^. The former does not agree with the experimental results, but the later does. The dispersion relation of the high-frequency turbulence coincides with both the $\omega ^2 = \Omega _\mathrm{ci}^2 + C_\mathrm {s}^2 k_\perp ^2 \approx C_\mathrm {s}^2 k_\perp ^2$ for the EICW or $\omega \approx k_\theta V_\mathrm {*} \approx k_\theta C_\mathrm {s}$ for the ICDW. EICW has been observed in linear plasmas^41,42^, and is driven by plasma current which have not been observed in the experiment. ICDW is a high-frequency branch of drift wave destabilized when the density gradient scale length is comparable to the ion inertial scale length^19,43^ and which agrees with the experimental results. Ion bernstein waves which have similar to the dispersion relation of the ICDW and EICW could be also one of the candidates. Distingishing the these instabilities requires plasma current measurement and is fruitful because both these instabilites could have important roles related to electron heat transport, plasma heating and energization in space plasmas^3,4^ and fusion plasmas^5,40,44^.

Finally, we discuss the broadening of the turbulent specturm. In general, ion-scale turbulence often has intermittency and have been discussed comparing with spectrum broadening^45–47^. The probability function analysis shows small skewness of about -0.1 which means the intermittency is no observed here. Typically, the intermittency of the turbulence may appear as power law in the spectrum. In our experiment, the spectrum broadening seems not to obey power law in observed frequency range. The other factors could be broadening the spectrum, for example the broadened growth rate itself by linearity or nonlinearity.

## Summary

In conclusion, we have achieved a high-spatial resolution observation of spatiotemporal dynamics of high-wavenumber turbulence in a basic laboratory plasma. The experiment was conducted aiming for only higher wavenumber fluctuations than the ion inertial scale length as $k_\theta \rho _\mathrm {s} > 1$ can be excited, controlled by a low magnetic field. As a result, low-frequency narrowband and high-frequency broadband fluctuations with $k_\theta \rho _\mathrm {s} \gg 1$ were identified. The autocorrelation function and spectral analyses revealed the correlation length of the high-wavenumber fluctuations, indicating that high-frequency broadband fluctuations have turbulent characteristics. The presented experiment is a pioneering study for the fundamental process of high-wavenumber turbulence in basic laboratory plasmas. The observations of spatiotemporal dynamics of high-wavenumber turbulence contribute to a comprehensive understanding of small-scale plasma turbulence and related nonlinear processes, anomalous transport, and heating.

## Methods

### Plasma Assembly for Nonlinear Turbulence Analysis (PANTA)

The experiments are conducted on PANTA, a linear laboratory plasma apparatus with a vacuum vessel 450 mm in diameter and 4000 mm long^48^. A homogeneous axial magnetic field ($B_\mathrm {ax}$) is applied to the plasma by 17 pairs of Helmholtz coils. Argon plasma with a diameter of 100 mm is produced by helicon wave heating (7MHz, 3kW) from the quarts tube with axial length of 400 mm and inner diamiter of 95 mm. Neutral gas pressure is controlled as about 0.2 Pa by solenoid valv. Under standard experimental conditions on the PANTA, the magnetic field is set above $B_\mathrm {ax}=$ 90 mT to study the nonlinear processes of ion-scale drift wave turbuelence ($k_\theta \rho _\mathrm {s}<1$) and mesoscale structure formation^11,25^. Drift wave turbulence experiments with a setting to the magnetic field near $B_\mathrm {ax}\sim$ 100 mT have also been studied vigorously in various laboratory plasmas^9,10,22^.

### Langmuir probe array

High spatiotemporal resolution measurement of plasma turbulence is realized by using an azimuthally aligned 64ch Langmuir probe array^21^. The 64ch probe array consists of 64 tungsten tips, arranged at equal intervals in the azimuthal direction at *r* = 40 mm, where *r* is the plasma radius, and is installed at 1885 mm away from the plasma source. It has a temporal resolution of $f_\mathrm {s}=$ 1 MHz and a spatial resolution of 3.9 mm, corresponding to an azimuthal wavenumber ($k_\theta$) resolution of $\delta k_\theta =$ 0.25/cm. The maximum measurable $k_\theta$ is $k_\theta =\pm$ 8/cm, restricted to the Nyquist theorem, or $k_\theta =$ 16/cm, assuming a propagation direction of fluctuations. The corresponding azimuthal mode numbers (*m*) are $m=$ 32 and $m=$ 64.

The density fluctuations are measured by each probe tip biased negative enough to collect ion saturation current. The electronic circuit of ion saturation current measurement is simple, consisting of a shunt resistor. The digital low pass filter 600kHz is applied to suppress the aliasing of the RF signal. In general, ion saturation current measurement is not cared about the details of the circuit because of the good frequency response. On the other hand, if we are measuring floating potential, we need to be careful. The existing systems with an electric circuit for floating potential have phase delays starting at about 20 kHz. Even if there is a phase delay in the measurement of ion saturation currents, it does not affect the wavenumber analysis because the same circuit is used for each ion saturation current measurement. However, it is necessary to compensate for the phase delay of floating potential measurement to evaluate fluctuation-induced particle transport.

### Two-dimensional space-time Autocorrelation function analysis

Autocorrelation function analysis is well-estabished within the plasma community. The definition is written as

1 $$\begin{aligned} C_1(\tau ) = \frac{1}{\sigma _\mathrm {t}^2}\frac{1}{T} \int _0^T f(t) f(t + \tau )dt, \end{aligned}$$

where $C_1$ is autocorrelation function, $\tau$ is time lag, $\sigma _t$ is standard deviation of zero-mean original time series of *f*(*t*), and *T* is data length in time. This study utilizes a two-dimensional autocorrelation function analysis that extends it to the temporal and spatial domains. The two-dimensional autocorrelation function $C_2$ is defined as

2 $$\begin{aligned} C_2(\tau , \xi ) = \frac{1}{\sigma _\mathrm {tx}^2}\frac{1}{TL} \int _0^L \int _0^T f(t, x) f(t + \tau , x + \xi ) dt dx, \end{aligned}$$

where $\xi$ is spatial lag, $\sigma _\mathrm {tx}$ is standard deviation of zero-mean original spatial and temporal image of *f*(*t*, *x*), and *L* is data length in space. We note that this analysis is different from the cross-correlation function analysis which analyzes the correlation between the reference signal and other signal. Typical, the cross-correlation function is used for data obtained in shot-by-shot manner^11^, sometimes the cross-correlation functions are applied to data obtained simultaneously^49,50^. The two-dimensional autocorrelation function is analyzed without one fixed signal as a reference signal but with its 2-dimensional images. It is compatible with data obtained from multi-point measurements that satisfy periodic boundary conditions, such as 64-ch probe arrays. The advantage of this analysis is to be able to capture the way turbulence is excited and decays during propagation.

## Data Availability

The datasets used and/or analysed during the current study available from the corresponding author on reasonable request.
